# Supplementary material for: Predicting susceptibility to tuberculosis based on gene expression profiling in dendritic cells
Source: Sci Rep. 2017 Jul 18;7:5702. doi: 10.1038/s41598-017-05878-w (PMC5516010; doi:10.1038/s41598-017-05878-w)
Supplement: Supplementary file 1 — Supplementary information [file 41598_2017_5878_MOESM1_ESM.pdf]

Supplementary information

Predicting susceptibility to tuberculosis based on gene expression profiling in dendritic cells

John D. Blischak[1,2,†], Ludovic Tailleux[3,†,*], Marsha Myrthil[1], Cécile Charlois[4], Emmanuel Bergot[5], Aurélien Dinh[6], Gloria Morizot[7], Olivia Chény[8], Cassandre Von Platen[8], Jean-Louis Herrmann[9,10], Roland Brosch[3], Luis B. Barreiro[11,12,*], Yoav Gilad[1,13,*]

[1]Department of Human Genetics, University of Chicago, Chicago, Illinois, USA [2]Committee on Genetics, Genomics, and Systems Biology, University of Chicago, Chicago, Illinois, USA [3]Integrated Mycobacterial Pathogenomics, Institut Pasteur, Paris, France [4]Centre de Lutte Antituberculeuse de Paris, DASES Mairie de Paris, 75013 Paris, France [5]Service de pneumologie et oncologie thoracique, CHU Côte de Nacre, 14033 Caen, France [6]Maladies Infectieuses, AP-HP, Hôpital Universitaire Raymond-Poincaré, Garches 92380, France [7]Clinical Investigation & Access Biological Resources (ICAReB), Institut Pasteur, Paris, France [8]Clinical Core, Centre for Translational Science, Institut Pasteur, Paris, France [9]INSERM, U1173, UFR Simone Veil, Université de Versailles Saint Quentin, Saint Quentin en Yvelines, France [10]APHP, Groupe Hospitalo-Universitaire Paris Île-de-France Ouest, Garches et Boulogne-Billancourt, France [11]Department of Genetics, CHU Sainte-Justine Research Center, Montreal, Québec, Canada [12]Department of Pediatrics, University of Montreal, Montreal, Québec, Canada [13]Department of Medicine, University of Chicago, Chicago, Illinois, USA

[†]These authors contributed equally.

[*]Correspondence should be addressed to Y.G. (gilad@uchicago.edu), L.T. (tailleux@pasteur.fr), and L.B.B. (luis.barreiro@umontreal.ca).

Supplementary figures

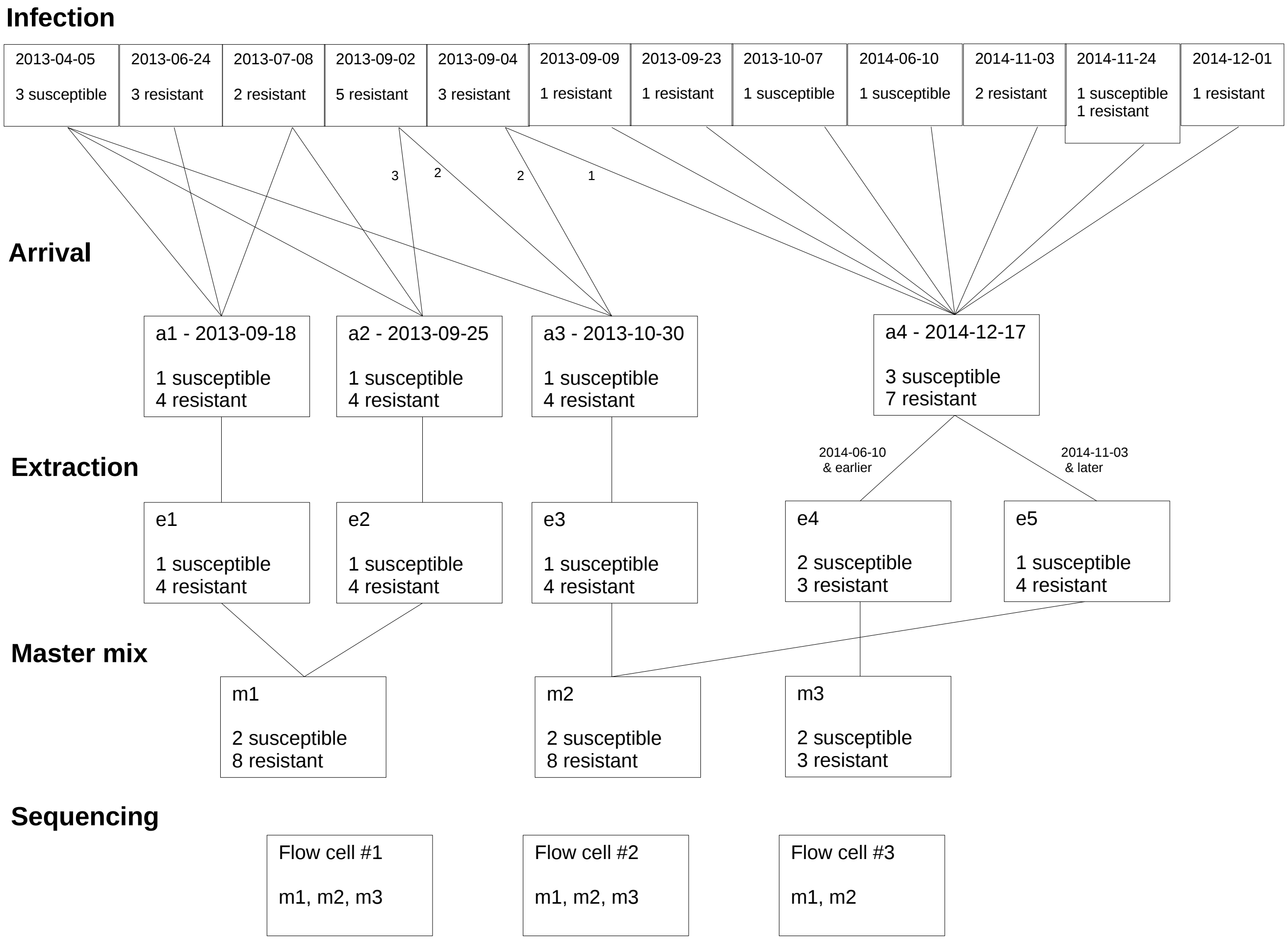

Figure S1. Batch processing. We designed the processing of the samples to minimize the introduction of technical batch effects. Specifically, we attempted to balance the processing of samples obtained from susceptible and resistant individuals. In the diagram, each box represents a batch. “Infection” labels the batches of the infection experiments, “Arrival” labels the batch shipments of cell lysates arrived in Chicago, USA from Paris, France, “Extraction” labels the batches of RNA extraction, “Master Mix” labels the batches of library preparation, and “Sequencing” labels the batches of flow cells. Each master mix listed in a flow cell batch was sequenced on only one lane of that flow cell.

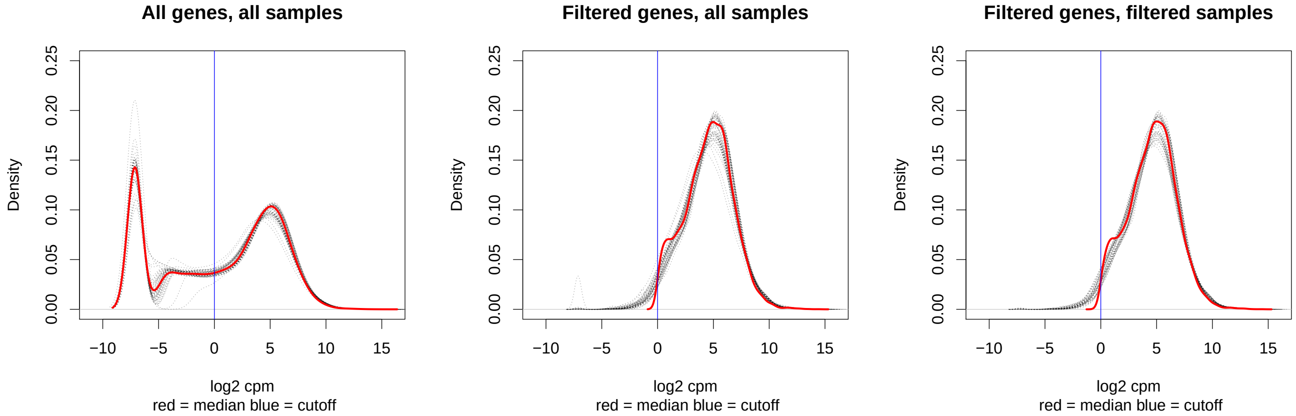

Figure S2. Gene expression distributions before and after filtering genes and samples. The $\log_2$ counts per million (cpm) of each sample is plotted as a dashed gray line. The solid red line represents the median value across all the samples. The vertical solid blue line at $x = 0$ represents the cutoff used to filter lowly expressed genes based on their median $\log_2$ cpm. The left panel is the data from all 19,800 genes and 50 samples, the middle panel is the data from the 11,336 genes remaining after removing lowly expressed genes, and the right panel is the data from 11,336 genes and the 44 samples remaining after removing outliers.

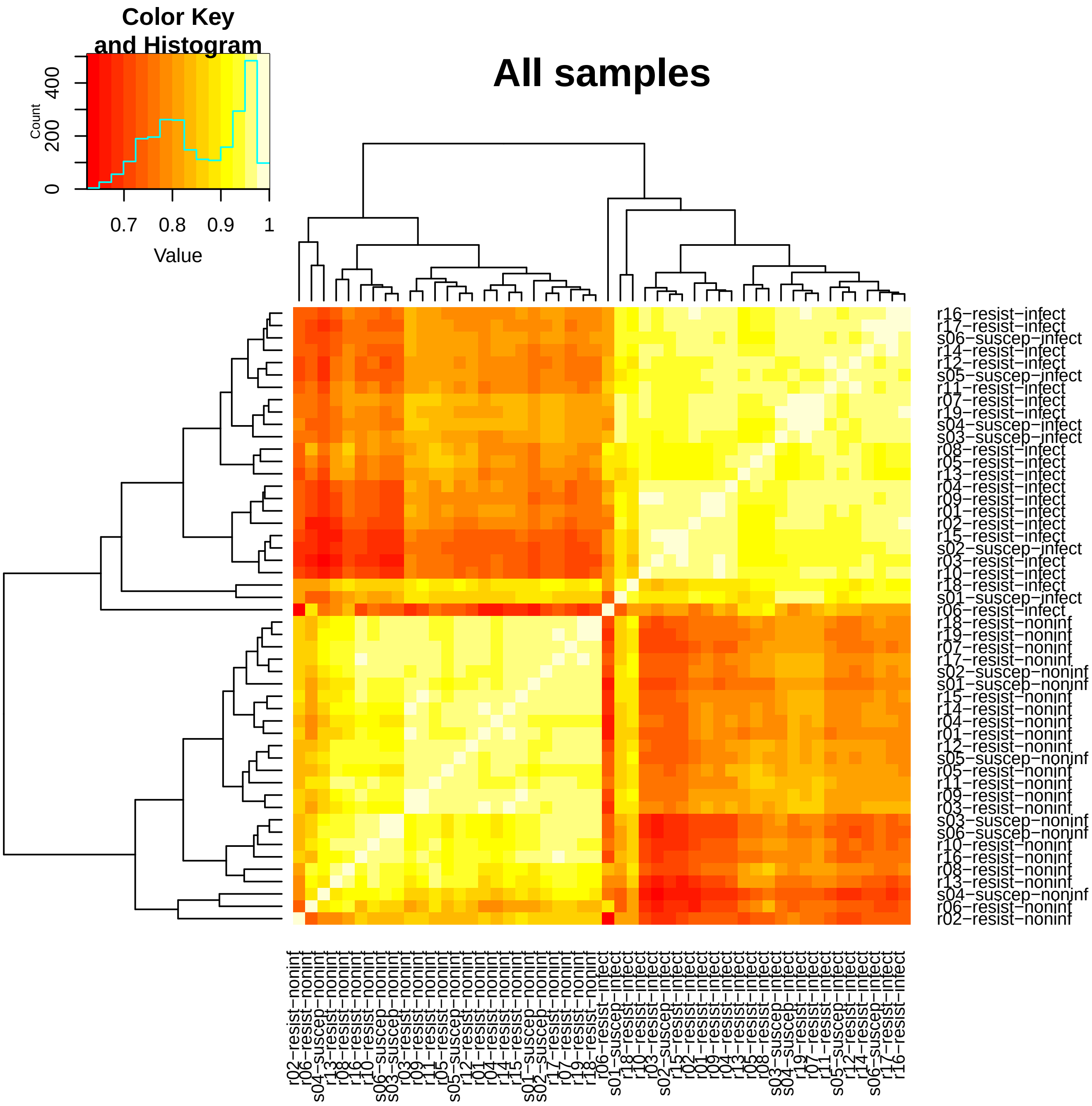

Figure S3. Heatmap of correlation matrix of samples. Each square represents the Pearson correlation between the $\log_2$ cpm expression values of two samples. Red indicates a low correlation of zero and white represents a high correlation of 1. The dendrogram displays the results of hierarchical clustering with the complete linkage method. The outliers of the non-infected samples are s04-suscept-noninf, r02-resist-noninf, and r06-resist-noninf. The outliers of the infected samples are s01-suscep-infect, r06-resist-infect, and r18-resist-infect.

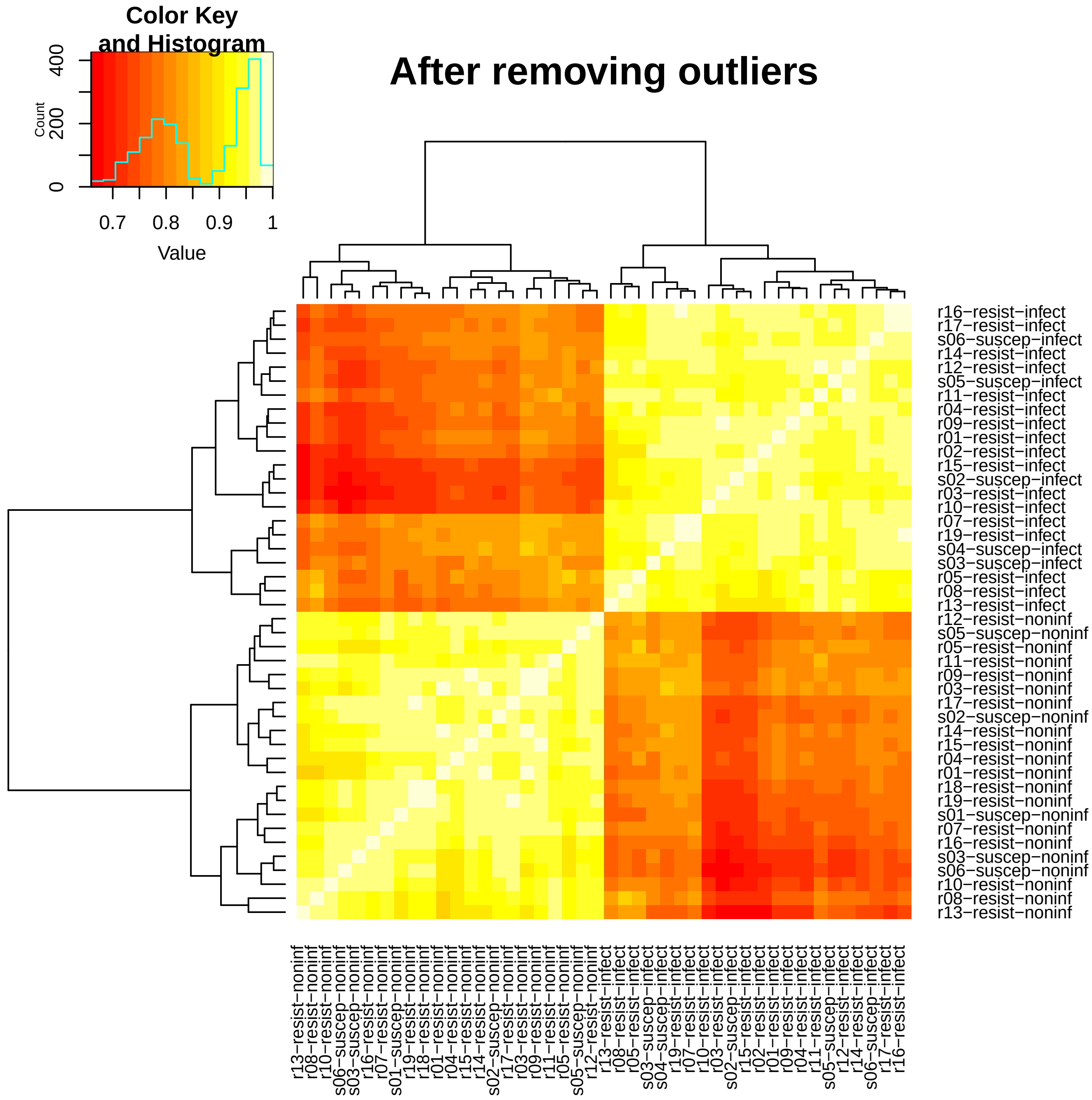

Figure S4. Heatmap of correlation matrix after removing outliers. Each square represents the Pearson correlation between the $\log_2$ cpm expression values of two samples. Red indicates a low correlation of zero and white represents a high correlation of 1. The dendrogram displays the results of hierarchical clustering with the complete linkage method.

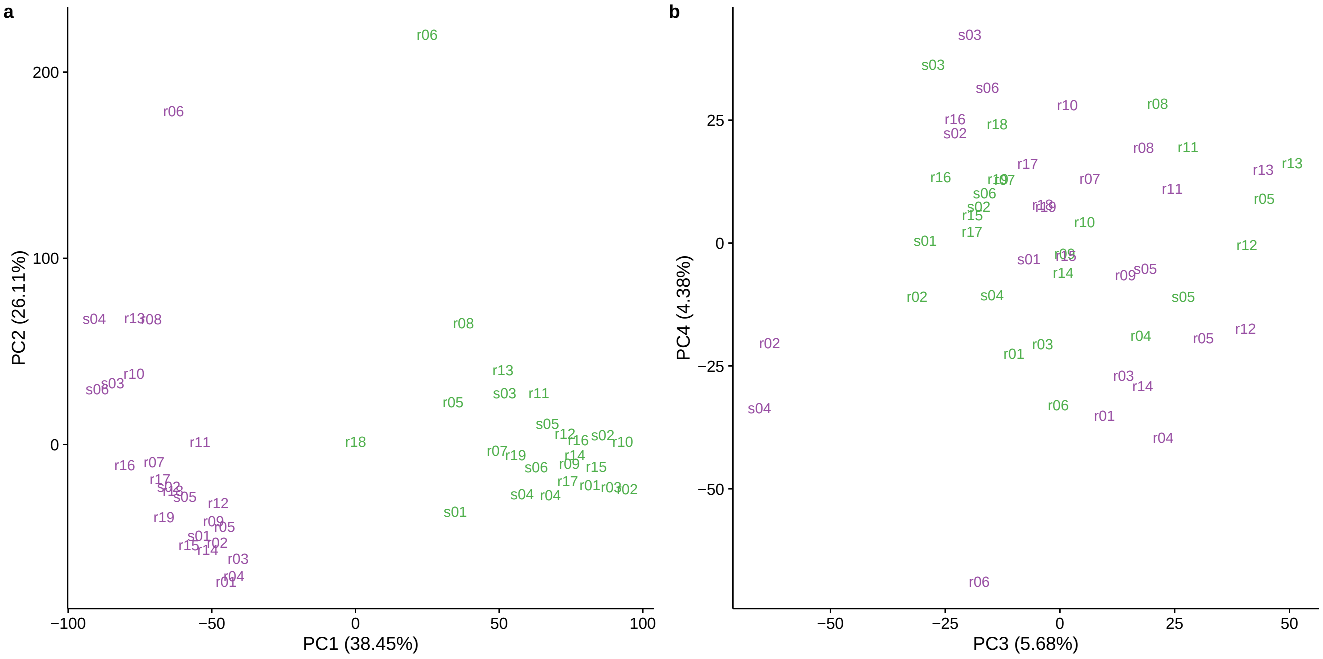

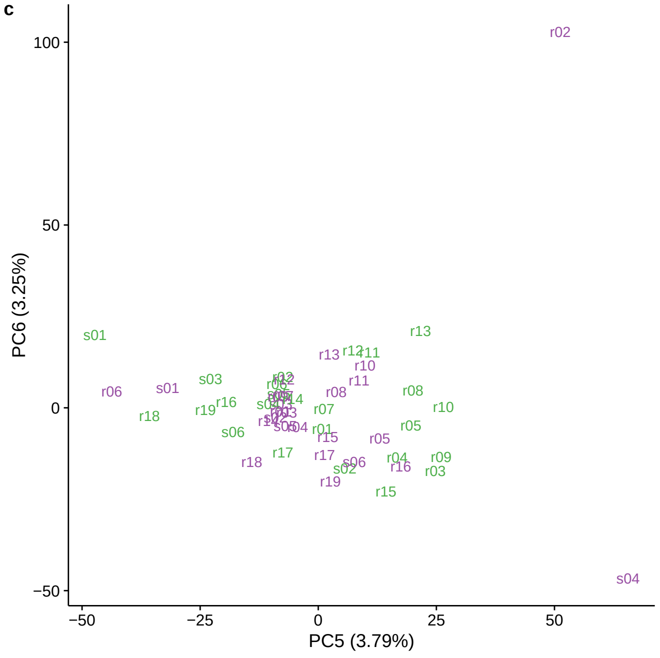

d

Sample	PC
r18−resist−infect	1
r06−resist−infect	2
r06−resist−noninf	2, 4, 5
r02−resist−noninf	3, 5, 6
s04−suscep−noninf	3, 5, 6
s01−suscep−infect	5

Figure S5. Principal components analysis (PCA) to identify outliers. (a) PC1 versus PC2, (b) PC3 versus PC4, and (c) PC5 versus PC6. Each sample is represented by its 3-letter ID. “s” stands for susceptible and “r” for resistant, and the text is colored on the basis of treatment status (purple is non-infected; green is infected). The value in parentheses in each axis is the percentage of total variation accounted for by that PC. The outliers are listed in (d). These samples do not fall within 2 standard deviations of the mean value of the PCs listed in the right column. Note that a separate mean was calculated for the non-infected and infected samples for PC1 only.

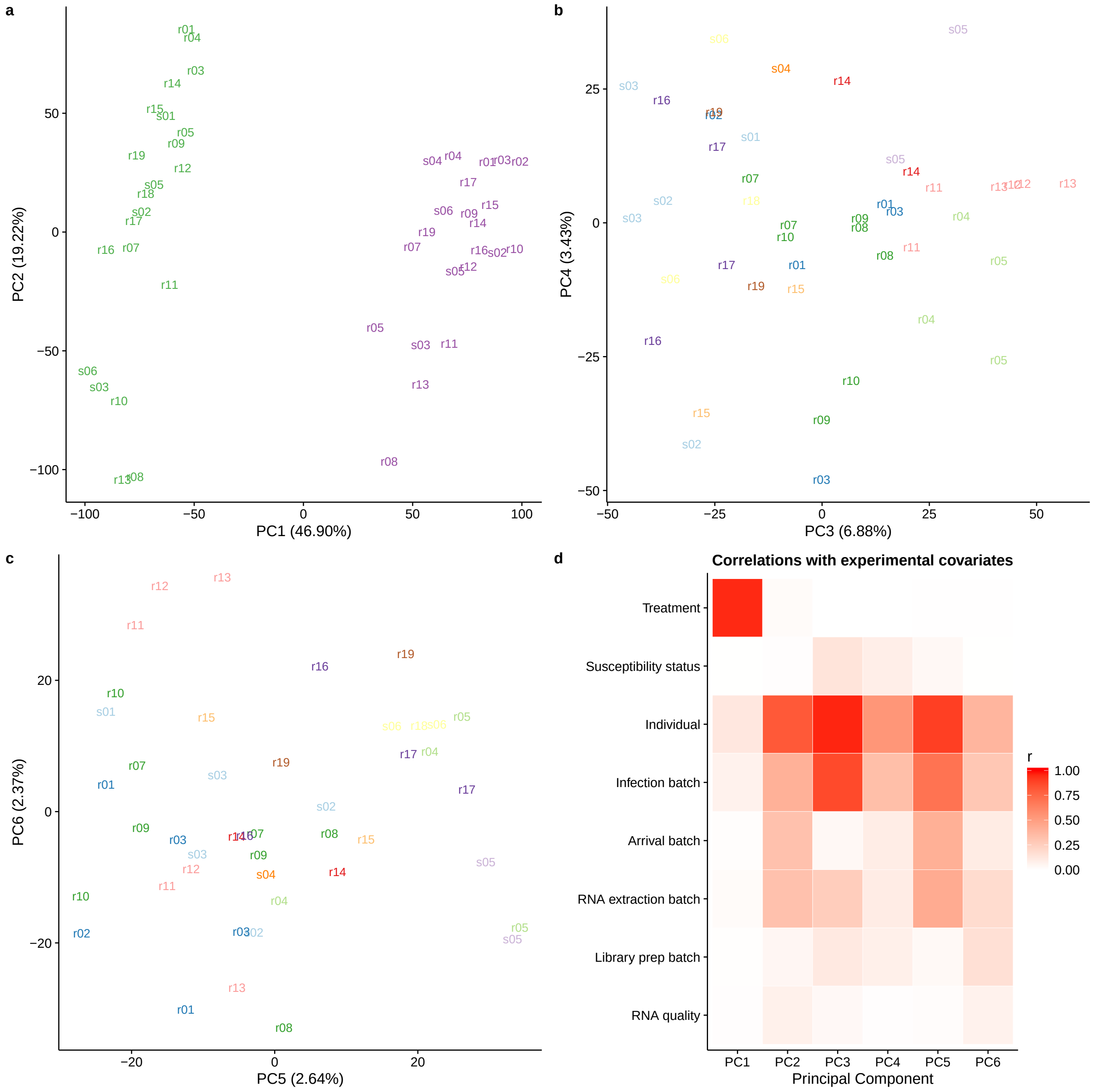

Figure S6. Check for technical batch effects using principal components analysis (PCA). (a) PC1 versus PC2. The text labels are the individual identifiers. Purple indicates non-infected samples and green indicates infected. (b) PC3 versus PC4. The colors indicate the different infection batches. (c) PC5 versus PC6. The colors indicate the different infection batches. (d) The Pearson correlation of PCs 1-6 with each of the recorded biological and technical covariates. The correlations vary from 0 (white) to 1 (red).

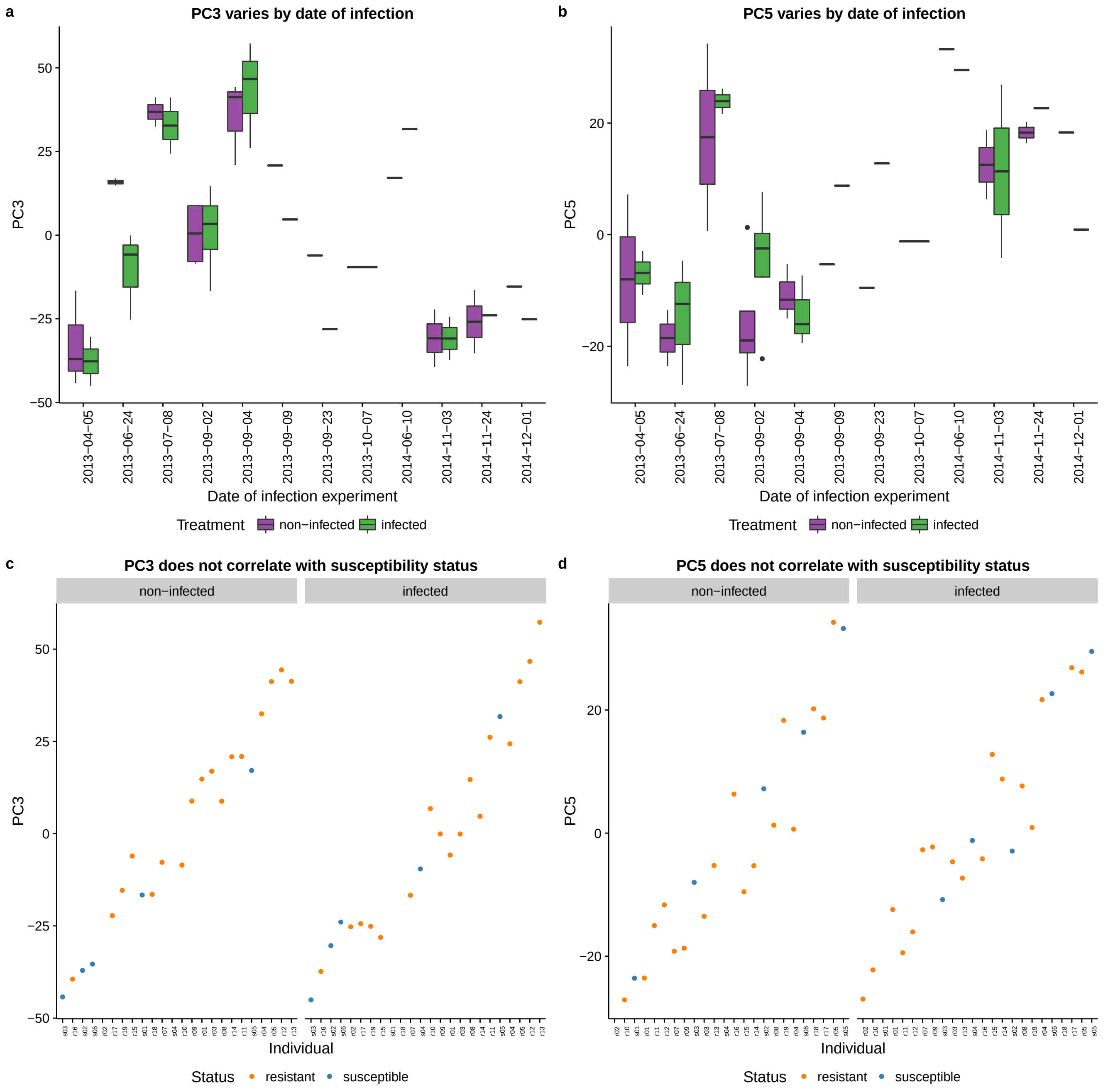

Figure S7. Check for confounding effect of infection batch. PC3 (a) and PC5 (b) varied by the date of infection. Non-infected samples are in purple and infected samples in green. Importantly, however, this technical variation arising from infection batch did not correlate with the susceptibility status of the individuals (c and d). Resistant individuals are in orange and susceptible individuals in blue.

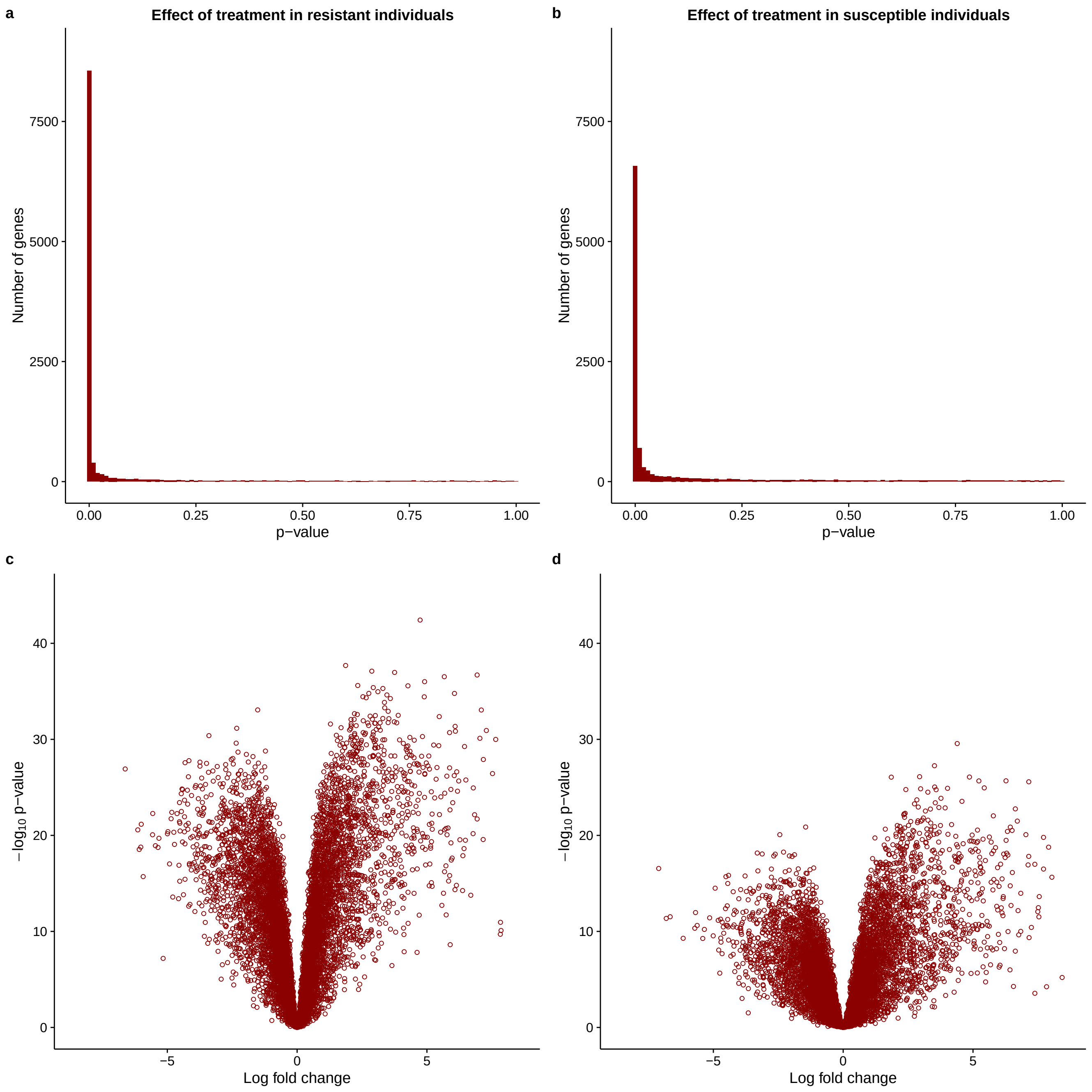

Figure S8. Effect of treatment with MTB. The top panel contains the distribution of unadjusted p-values after testing for differential expression between the non-infected and infected states in (a) resistant and (b) susceptible individuals. The bottom panel contains the corresponding volcano plots for the (c) resistant and (d) susceptible individuals. The x-axis is the log fold change in gene expression level between susceptible and resistant individuals and the y-axis is the $-\log_{10}$ p-value. Red indicates genes which are significant differentially expressed with a q-value less than 10%. Because of the extremely skewed p-value distribution, all genes are significantly differentially expressed at this false discovery rate.

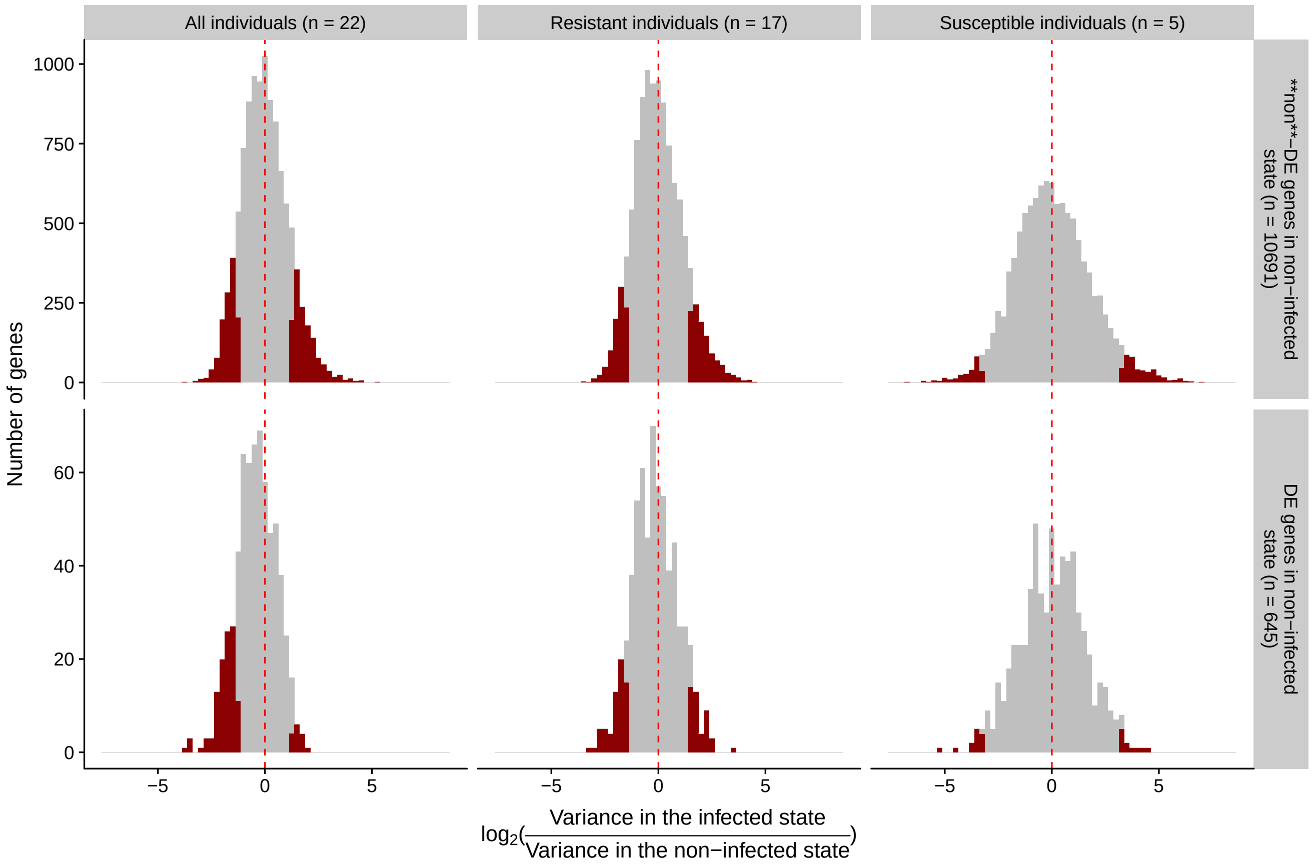

Figure S9. Check for systematic differences in gene expression variance between the infected and non-infected states. We identified DE genes between susceptible and resistant individuals in the non-infected but not the infected state. Could this be a statistical artifact due to an overall increase in gene expression variance upon infection thus reducing power to detect DE genes? No, because we did not observe an overall increase in gene expression variance in the infected state. The histograms show the distribution of the $\log_2$-transformed ratio of the gene expression variance in the infected state to the variance in the non-infected state. If there was an overall increase in variance, the distributions should be shifted towards the right, but instead they are all symmetrical. The top row shows the results for the 10,691 genes which were not differentially expressed between susceptible and resistant individuals in the non-infected state, and the bottom row shows the results for the 645 genes which were. The left column shows the results for all 22 individuals in the study, the middle column for the 17 resistant individuals, and the right column for the 5 susceptible individuals (note that the right column has the widest spread because of this small sample size). Highlighted in red are genes which had a $P < 0.05$ from an F test comparing the two variances. The number of genes with a significant increase or decrease in variance was also mostly symmetrical (decrease vs. increase starting at top left panel and proceeding clockwise: 1,232 vs. 1,362; 934 vs. 1,118; 275 vs. 455; 13 vs. 11; 64 vs. 44; 108 vs. 15).

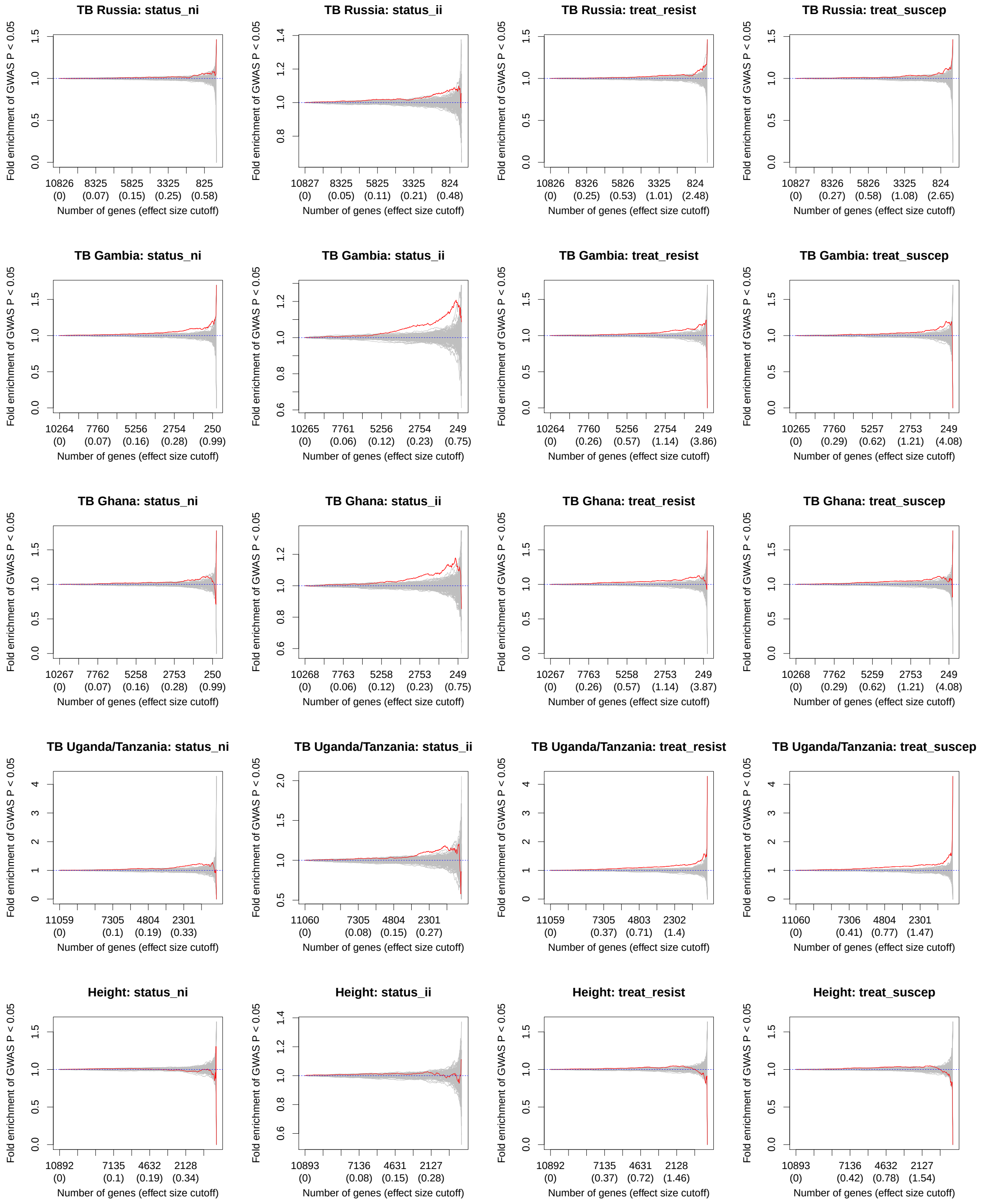

Figure S10. Comparison of differential expression and GWAS results. In each subplot, the y-axis is the fold enrichment (y-axis) of genes assigned a SNP with p-value less than 0.05 from the GWAS. The x-axis is bins of genes with increasingly stringent effect size cutoffs of the absolute log fold change for the different expression contrast. The effect size cutoffs were chosen such that each bin from left to right contained approximately 25 fewer genes. The red line is the results from the actual data. The grey lines are the results from 100 permutations. The dashed blue line at y=1 is the null expectation. The rows correspond to the 5 GWAS studies: Russia[18], The Gambia[13], Ghana[13], Uganda and Tanzania[19], and height in individuals of European ancestry[25]. The columns correspond to the 4 differential expression contrasts: resistant vs. susceptible individuals in the non-infected state (status_ni), resistant vs. susceptible individuals in the infected state (status_ii), effect of treatment in resistant individuals (treat_resist), and effect of treatment in susceptible individuals (treat_suscep). The x-axis slightly varies based on the number of genes that were able to be assigned a nearby SNP for each GWAS, and thus is consistent only within each study (i.e. row, although the exact tick labels in each plot slightly vary based on R's rules for annotating axes). The y-axis is set separately for each plot based on the minimum and maximum fold enrichment values for that particular analysis.

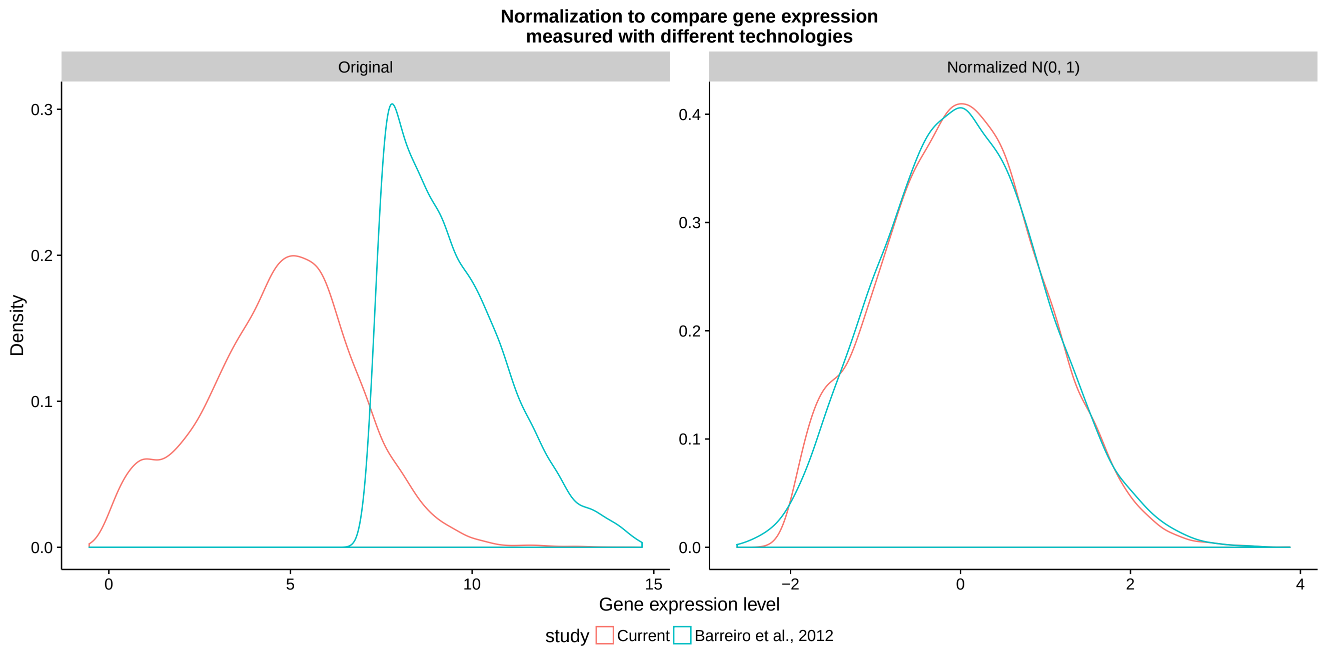

Figure S11. Normalizing gene expression distributions. (left) The distribution of the median log2 cpm of the RNA-seq data from the current study in red compared to the distribution of the median gene expression levels of the microarray data from Barreiro et al., 2012[24] in blue. (right) The distributions of the same data sets after normalizing each sample to a standard normal distribution.

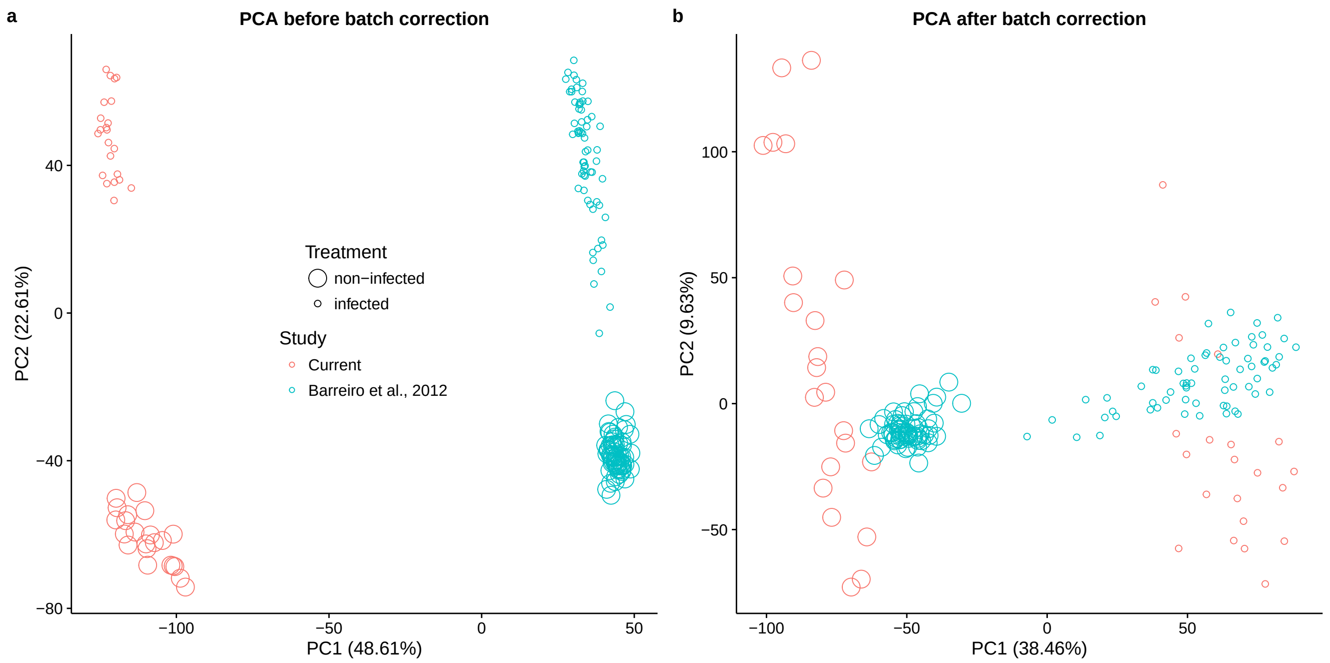

Figure S12. Principal components analysis (PCA) of combined data sets. (a) PC1 versus PC2 of the combined data set of the RNA-seq data from the current study (red) and the microarray data from Barreiro et al., 2012[24] (blue). The large circles are non-infected samples, and the small circles are infected samples. The value in parentheses is the percentage of the total variation accounted for by that PC. (b) The same data after regressing the original PC1 in (a).

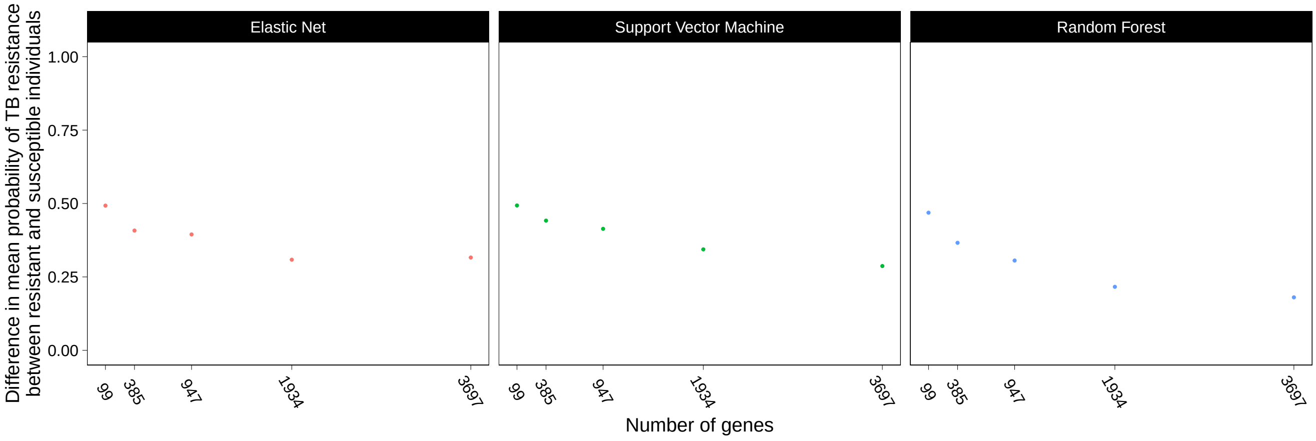

Figure S13. Comparing the classification results of different methods and number of input genes. We compared 3 different machine learning methods (elastic net, support vector machine, random forest) and used 5 different sets of input genes. The input genes (x-axis) were obtained by varying the q-value cutoff for differential expression between susceptible and resistant individuals in the non-infected state from 5% to 25%. The evaluation metric (y-axis) was the difference of the mean assigned probability of being TB resistant between the known resistant and susceptible individuals in the current study.

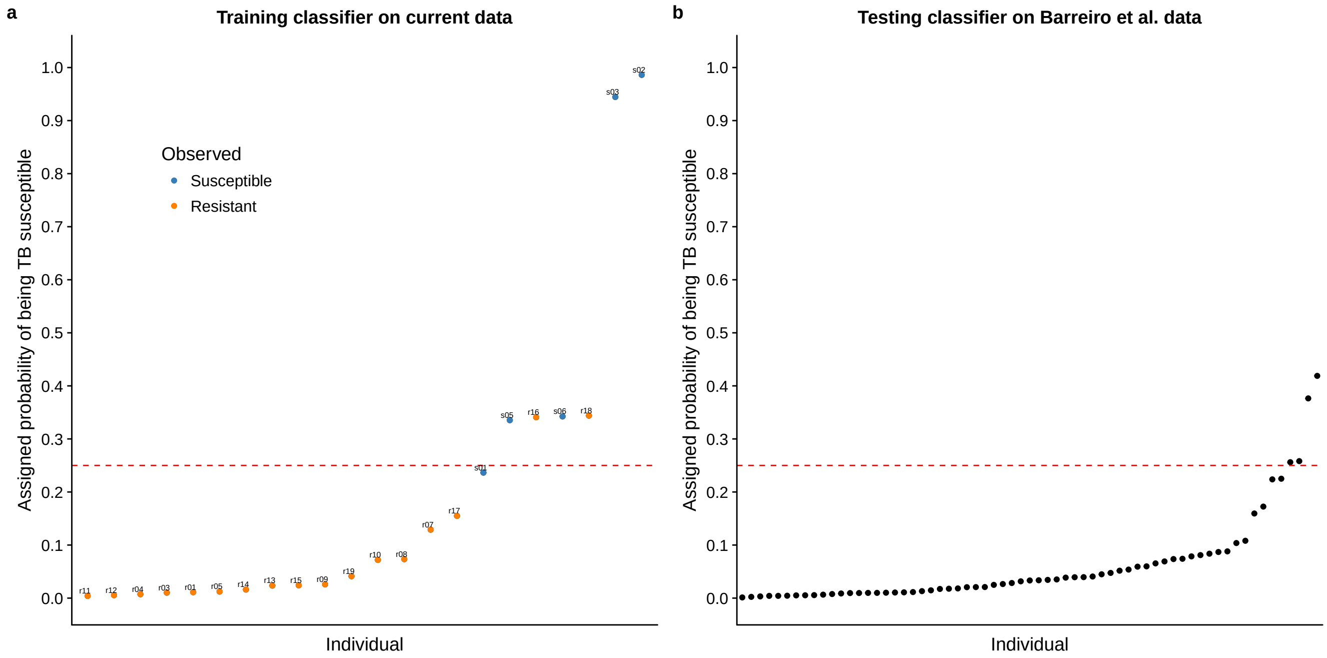

Figure S14. Classifying TB susceptible individuals using an elastic net model. (a) The estimates of predicted probability of TB susceptibility from the leave-one-out-cross-validation for individuals in the current study. The blue circles represent individuals known to be susceptible to TB, and orange those resistant to TB. The horizontal blue line at a probability of 0.25 almost separates susceptible and resistant individuals. (b) The estimates of predicted probability of TB susceptibility from applying the classifier trained on the data from the current study to a test set of independently collected healthy individuals[24].

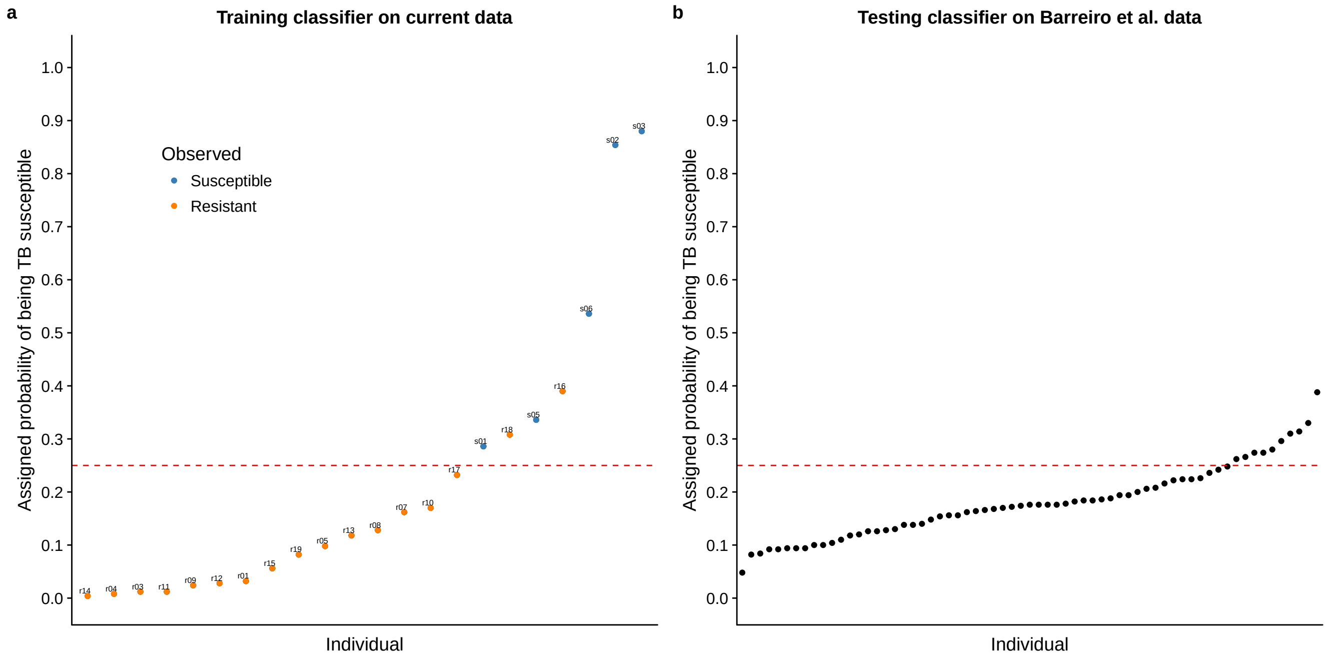

Figure S15. Classifying TB susceptible individuals using a random forest model. (a) The estimates of predicted probability of TB susceptibility from the leave-one-out-cross-validation for individuals in the current study. The blue circles represent individuals known to be susceptible to TB, and orange those resistant to TB. The horizontal blue line at a probability of 0.25 separates susceptible and resistant individuals. (b) The estimates of predicted probability of TB susceptibility from applying the classifier trained on the data from the current study to a test set of independently collected healthy individuals[24].

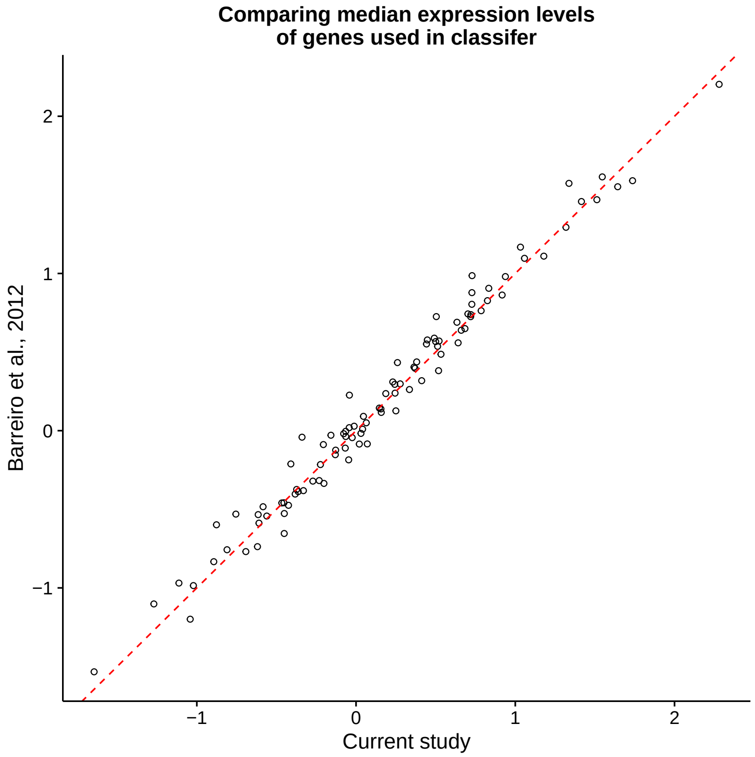

Figure S16. Comparing gene expression between the two studies. After normalization and batch-correction, the median expression levels of the 99 genes used in the classifier were similar between the samples in the current study and those in Barreiro et al., 2012[24]. The dashed red line is the 1:1 line.

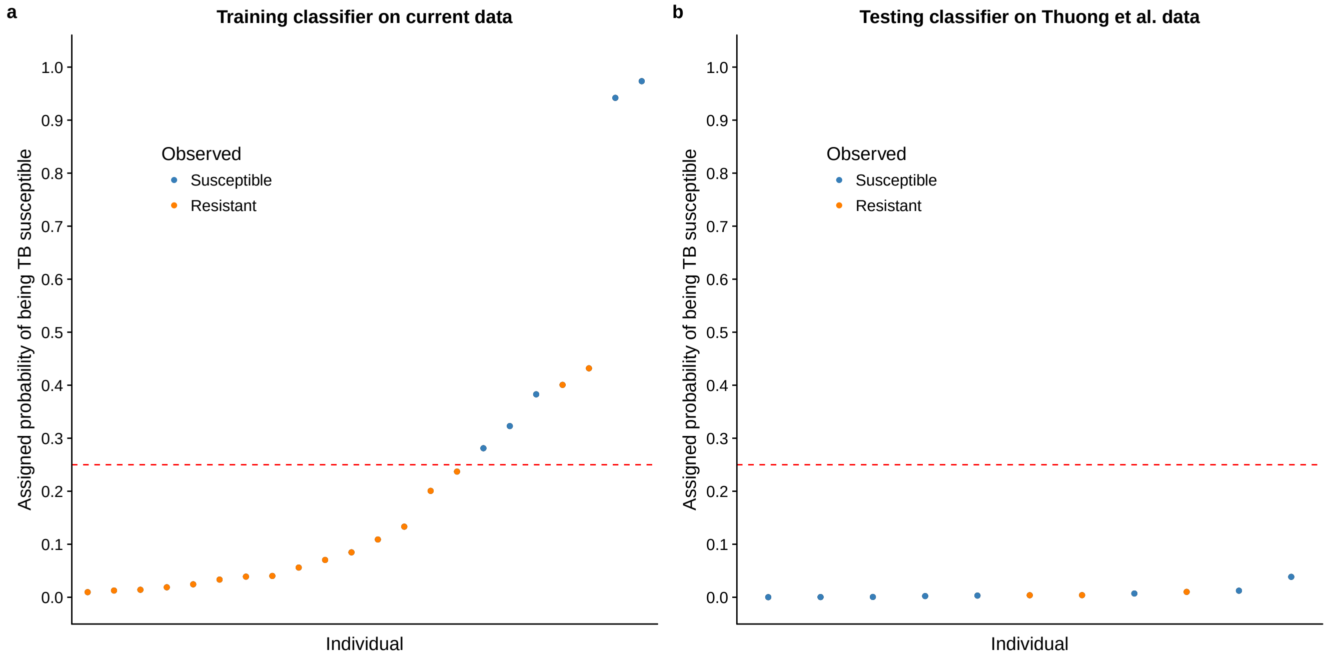

Figure S17. Classifying individuals from Thuong et al., 2008[20] using a support vector machine model. We followed the same training and testing procedure performed for testing the classifier described in the main text (Fig. 3, see Classifier in Methods). Not surprisingly since the data sets were from different cell types, the classifier trained on the dendritic cells in this study performed poorly when tested on samples with gene expression levels measured in macrophages. To match our naming system, we labeled the individuals from Thuong et al., 2008[20] with latent TB as resistant (n = 3 after removing the outlier sample LTB2) and the individuals recovered from pulmonary or meningeal TB as susceptible (n = 4 each). (a) The estimates of predicted probability of TB susceptibility from the leave-one-out-cross-validation for individuals in the current study. The blue circles represent individuals known to be susceptible to TB, and orange those resistant to TB. The horizontal dashed red line at a probability of 0.25 separates susceptible and resistant individuals. (b) The estimates of predicted probability of TB susceptibility from applying the classifier trained on the data from the current study to a test set of putatively susceptible and resistant individuals[20].

Supplementary data

Supplementary Data S1

Supplementary Data S1 contains information on the 50 samples. Most variables describe the batch processing steps outlined in Supplementary Fig. S1. “id” is a unique identifier for each sample, “individual” is the individual identifier (“s” = susceptible, “r” = resistant), “status” is the susceptibility status, “treatment” is if the sample was infected or non-infected, “infection” is the date of the infection experiment (12 total), “arrival” is the identifier for the arrival batch (4 total), “extraction” is the batch for RNA extraction (5 total), “master_mix” is the batch for library preparation (3 total), “rin” is the RNA Integrity Number from the Agilent Bioanalyzer, and “outlier” is a Boolean variable indicating if the sample was identified as an outlier (Supplementary Fig. S5) and removed from the analysis. (tds)

Supplementary Data S2

Supplementary Data S2 contains the gene expression counts for the 11,336 genes after filtering lowly expressed genes for all 50 samples (Supplementary Fig. S2). Each row is a gene labeled with its Ensembl gene ID. Each column is a sample. Each sample is labeled according to the pattern “x##-status-treatment”, where x is “r” for resistant or “s” for susceptible, ## is the ID number, status is “resist” for resistant or “suscep” for susceptible, and treatment is “noninf” for non-infected or “infect” for infected. (tds)

Supplementary Data S3

Supplementary Data S3 contains the results of the differential expression analysis with limma (Fig. 1). The workbook contains 4 sheets corresponding to the 4 tests performed. “status_ni” is the test between resistant and susceptible individuals in the non-infected state, “status_ii” is the test between resistant and susceptible individuals in the infected state, “treat_resist” is the test between the non-infected and infected states for resistant individuals, and “treat_suscep” is the test between the non-infected and infected states for susceptible individuals. Each sheet has the same columns. “id” is the Ensembl gene ID, “gene” is the gene name, “logFC” is the log fold change from limma, “AveExpr” is the average log expression from limma, “t” is the t-statistic from limma, “P.Value” is the p-value from limma, “adj.P.Val” is the adjusted p-value from limma, “qvalue” is the q-value calculated with adaptive shrinkage, “chr” is the chromosome where the gene is located, “description” is the description of the gene from Ensembl, “phenotype” is the associated phenotype(s) assigned my Ensembl, “go_id” is the associated GO term(s) assigned by Ensembl, and “go_description” is the corresponding name(s) of the GO term(s). (xlsx)

Supplementary Data S4

Supplementary Data S4 contains the results of the GWAS comparison analysis (Fig. 2). The first sheet “input-data” contains the p-values for the GWAS SNP assigned to each gene from each study. The columns “gwas_p_russia”, “gwas_p_gambia”, “gwas_p_ghana”, “gwas_p_uganda”, “gwas_p_height” contain the p-values from the TB susceptibility GWAS in Russia, The Gambia, Ghana, Uganda and Tanzania, and the height GWAS in Europeans, respectively. The columns “status_ni”, “status_ii”, “treat_resist”, and “treat_suscep” refer to the tests described for Supplementary Data S3 and contain the log fold changes for each comparison. All the other gene annotation columns are the same as described for Supplementary Data S3. The second sheet “top-genes” contains a subset of the full results to highlight those genes which had an absolute log fold change greater than 2 between resistant and susceptible individuals in the non-infected state (“status_ni”). (xlsx)

Supplementary Data S5

Supplementary Data S5 contains the results of the classifier analysis. Specifically it contains the results from the support vector machine using the genes with a q-value less than 0.05 (Fig. 3). The sheet “gene-list” contains information about the genes used for the classifier (the columns are described in the section for Supplementary Data S3). The sheet “training-input” contains the input gene expression data for training the model. The sheet “training-results” contains the results of the leave-one-out-cross-validation when training the model on the samples from the current study. The sheet “testing-input” contains the input gene expression data for testing the model. The sheet “testing-results” contains the results from testing the model on the samples from Barreiro et al., 2012[24]. The column “prob_tb_suscep” is the probability of being susceptible to TB assigned by the model. (xlsx)
